# EGLN1 induces tumorigenesis and radioresistance in nasopharyngeal carcinoma by promoting ubiquitination of p53 in a hydroxylase-dependent manner

**DOI:** 10.7150/jca.66080

**Published:** 2022-03-28

**Authors:** Lu Sun, Cheng Wu, Jun Ming, Ergang Guo, Wei Zhang, Lingling Li, Guoqing Hu

**Affiliations:** 1 Department of Oncology, Tongji Hospital, Tongji Medical College, Huazhong University of Science and Technology, Wuhan, Hubei Province, China.; 2 Department of Radiation Cancer, Hubei Cancer Hospital, Tongji Medical College, Huazhong University of Science and Technology, Wuhan, China.; 3 The Comprehensive Cancer Centre of Nanjing Drum Tower Hospital, The Affiliated Hospital of Nanjing University Medical School & Clinical Cancer Institute of Nanjing University, Nanjing, China.

**Keywords:** nasopharyngeal carcinoma, Egl-9 family hypoxia inducible factor 1, tumor protein p53, ubiquitination, cancer stem cell

## Abstract

Egl-9 Family Hypoxia Inducible Factor 1 (EGLN1) is a proline hydroxylase mediating degradation of hypoxia-inducible factor α (HIFα) through the ubiquitination system. Studies have indicated an essential role for EGLN1 in angiogenesis and tumorigenesis. However, there is no consensus on the regulation of EGLN1 and its mechanism of action on nasopharyngeal carcinoma (NPC). This study explored the association of the expression of EGLN1 with characteristics of NPC tumors and its underlying mechanism. We found that the expression of EGLN1 showed a positive correlation with tumor T classification and clinical staging of patients with NPC. EGLN1 could promote cell proliferation, invasion and migration, and even enhance the cancer stem cells (CSCs) prosperity and radioresistance of NPC cells. Mechanistically, EGLN1 facilitated degradation of tumor protein p53 through the ubiquitination system. This effect could be weakened in the presence of dimethyloxalylglycine (DMOG), suggesting that EGLN1 down-regulated p53 based on its hydroxylase activity. In conclusion, overexpression of EGLN1 promoted oncogenesis and induced a CSC-like phenotype in NPC cells, then enhancing the ability for radioresistance by interacting with p53 in a hydroxylase-dependent manner. Thus, EGLN1 might serve as a potential therapeutic target for NPC.

## Introduction

Nasopharyngeal carcinoma (NPC) is a malignancy commonly in Southeastern Asia, including Southern China 1. Intensity-modulated radiotherapy (IMRT) is frequently used in the treatment of NPC 2. Despite the improvement in chemoradiotherapy (CRT) technology and equipment, the prognosis of advanced NPC remains poor. The 5-years survival rate of patients with NPC is less than 30 % 3. The development of resistance to radioactive rays and antitumor drugs appears to be the main reason for therapy failure and metastasis. Therefore, understanding the molecular mechanism of tumor initiation and radioresistance in NPC could undoubtedly improve our ability to predict the response of NPC to radiotherapy and determine which subset of patients would benefit from therapy 4.

Egl-9 Family Hypoxia Inducible Factor members (EGLN1, 2 and 3), also called Prolyl Hydroxylase Domain (PHD2, 1 and 3), have been proposed to act as oxygen sensors 5. Their enzymatic activity has been shown to be depend on the presence of ascorbic acid, iron, oxygen and α-ketoglutarate 6. Mutations of EGLNs have been reported in various tumors, including lung cancer, and endometrial carcinoma 7,8. The expression of EGLNs was lower in patients with colorectal carcinoma than the healthy controls. Silencing of EGLN1 could activate the downstream pathway of hypoxia-inducible factor 1α (HIF1α) and Wnt/β-catenin, thus increasing the expression of cancer stem cell (CSCs) associated genes 9,10. In squamous cell cancer of the head and neck (HNSCC), high expression of EGLN1, and particularly high nuclear expression was shown to predict an unfavorable response to radiotherapy (RT) 11. In patients with classical Hodgkin's lymphoma, strong expression of EGLN1 was linked to short progression-free survival (PFS) and might also had a role in the resistance to first-line treatment 12.

It is worth noting that, EGLN1 has been widely reported to cause the increased ubiquitination and proteasomal degradation of hypoxia-inducible factor α (HIFα). It has been reported that the hydroxylation of prolyl residues 402 and 564 of HIFα by EGLN1 led it to be recognized by von Hippel-Lindau (VHL) E3 ligase complex for protein proteasomal degradation 13-14. On the other hand, loss of EGLN1 has been demonstrated to result in an accumulation of HIFα under normoxia or hypoxia. Recent studies have shown that EGLNs also have other substrates, except for HIFα. For example, Guo et al., found that EGLN1 prolyl-hydroxylated protein kinase B (PKB), also known as Akt on the oxygen-dependent way, has a critical role in tumor growth and therapeutic resistance 15. In addition, EGLN3 was shown to function as an inhibitor of the IKK/NF-κB signaling pathway, enhancing the therapeutic responsiveness of colorectal cancer cells 16.

Tumor protein p53 is known as the guardian of the genome and has demonstrated an association with the plasticity of cancer cells in oncogenesis, self-renewal, differentiation and reprogramming. Plenty of experimental evidence have indicated that loss or mutation of p53 leads to cell de-differentiation and acquisition of stemness 17,18. Furthermore, it was reported that p53 could be regulated by both EGLN2 and EGLN3. Accordingly, it was shown that EGLN2 decreased the half-life of p53 protein and negatively regulated NF-κB and p53 signaling pathways by hydroxylation of the proline 142 residue of p53 19. In contrast, EGLN3 was reported to reduce the degradation of p53 in colon cancer stem cells 20. Hence, the regulation of p53 by EGLNs is controversial, but most studies have confirmed that this regulatory function was related to the ability of EGLNs for hydroxylation. Furthermore, there have been no evidence so far, that EGLN1 is involved in the regulation of p53 and tumor initiation in NPC.

In this study, we detected the role of EGLN1 in the oncogenesis of NPC and response to radiotherapy of clinical-stage NPC, and explored its internal mechanisms. In our exploration, we demonstrated that high expression of EGLN1 predicted high T classification and clinical stage of patients with NPC. The overexpression of EGLN1 could promote cell proliferation, invasion, migration and enhance the properties of CSCs in NPC cells. In addition, EGLN1 could lead to increased cell radioresistance. Further inquiring into the mechanism, we confirmed the interaction of EGLN1 with p53, thereby promoting the ubiquitination enzyme system-mediated destruction of p53 and reducing the activation of the p53 pathway in a hydroxylase-dependent manner. Our findings revealed the mechanism underlying the regulation of p53 by EGLN1 and uncovered the role of EGLN1 in cancer progression. Therefore, targeting EGLN1 might have therapeutic significance in NPC in the future.

## Materials and methods

### Clinical tissues

Non-tumor nasopharyngeal epithelium and NPC samples were collected from the Department of Pathology, Tongji Hospital, Tongji Medical College, Huazhong University of Science and Technology between January 2015 and August 2018. Inclusion criteria: (1) Patients were confirmed by pathological examination. (2) The clinical data were complete. Patients were re-staged according to the AJCC 8th Cancer Staging Manual. (3) Age ≥16 years old. Exclusion criteria: (1) Patients who have received anti-tumor treatment before, including chemotherapy, radiotherapy, immunotherapy, targeted therapy and so on. (2) Patients with concurrent or previous diagnosis of other malignancies. (3) Patients with other life-threatening chronic diseases. According to the criteria, we collected a total of 124 cases (including 86 males and 38 females). At the same time, 10 pathological sections of patients with nasal polyps were randomly selected as the control group. All patients were diagnosed by two pathologists.

### Reagents

Primary antibodies used were as follows: Anti-Flag (#14793), anti-HA-Tag (#3724), anti-GAPDH (#5174), anti-P53 (#32532), anti-Nanog (#8822), anti-SOX2 (#3579), anti-Caspase 3 (#9662), anti-CD133 (#64326), anti-CD44 (#3570) and anti-EGLN1 (#4835) for WB were purchased from Cell Signaling Technology (USA). Anti-EGLN1 (#NB100-2219) for IHC was obtained from Novus Biologicals (USA). Anti-Bax (#50599‐2‐Ig) and anti-Bcl-2 (#12789‐1‐AP) was obtained from Protein Technology (Wuhan, China). Anti-Ub (sc-8017) were purchased from Santa Cruz Biotechnology (CA, USA). Anti-Flag (F1804) were purchased from Sigma Aldrich (USA). Dimethyloxalylglycine (DMOG), carbobenzoxy-Leu-Leu-leucinal (MG132), and cycloheximide (CHX) were purchased from Medchemexpress (USA).

### Immunohistochemistry

Unstained paraffin sections were dewaxed by incubation with varying concentrations of dimethylbenzene. Then, tissues were stained with EGLN1 antibody according to protocols. The staining score of EGLN1 was independently evaluated according to staining intensity and positive areas by 2 histopathologists. Staining intensity ranged from 0 (no staining), 1 (weakly positive), 2 (medium positive), to 3 (strongly positive). Positive areas were scored as follows: 1 (0-25%), 2 (25-50%), 3 (50-75%), and 4 (≥75%). The staining score was calculated by multiplying these 2 scores. A score <6 was defined as low expression, whereas as high expression.

### Cell culture

The NPC cell lines used in this study were purchased from the Cancer Research Institute of Central South University (Changsha, China). NP69 is a normal NPC mucosal epithelial cell. CNE1 is a well differentiated squamous cell carcinoma line of NPC; CNE2, HNE1 and HONE1 are poorly differentiated squamous cell carcinoma line of NPC; HONE1 a well differentiated squamous cell carcinoma line of NPC with EB virus positive. Cells were cultured in RPMI‐1640 medium (KeyGEN, Jiangsu, China) with 10% fetal bovine serum (FBS; Gibco Life Technologies, Grand Island, NY, USA). All cells were grown at 37 °C in 5 % CO_2_.

### Cell proliferation and invasion assay

The proliferation ability of cells was evaluated using the CCK8 assay (MedChemExpress, Shanghai, China) following the manufacturer's protocol. The cell scratch assay was used to evaluate the ability of cells for migration. Images were captured at 0 and 24 h. For the invasion assays, cells were plated in FBS-free medium in the upper chamber (Corning, Corning, NY) using a Matrigel, with the lower chamber filled with complete medium. After incubation for 24 h, cells were fixed and stained with methanol and crystal violet, respectively. Consecutively, invasion cells were counted under an upright microscopy.

### Colony formation assay

When cells reached about 80% confluence, they were diluted into single-cell suspension, and after dilution to the corresponding concentration, they were planted in 6-well plates. Each group was repeated 3 times. Then, cells were irradiated with 2-10 Gy doses and cultured for 10-14 d. After fixed with 4% paraformaldehyde, cells were stained with 0.1% crystal violet. Cells were observed under a microscope, more than 50 cells were counted as 1 clone. Clones were counted and analyzed using Photoshop CC 2019. Each experiment was independently repeated 3 times.

### Lentiviral transfection

The shELGN1, Flag-EGLN1-overexpression (EGLN1-OE), Flag-p53-overexpression (p53-OE), HA-RPS27A-overexpression lentiviral particles and the corresponding empty-vector were purchased from GeneChem (Shanghai, China). Lentiviruses were added into cultured cells according to the protocol. After being screened by puromycin, stably transfected cells were tested by western blot analysis.

### Western blotting assay

Cells were lysed by RIPA lysis buffer with 1% PMSF and 1 % protease inhibitor cocktail. Protein was obtained by centrifugation at 4 °C, 12 000 *g* for 15 min. The concentration of protein was measured by Nanodrop 2000 (Thermo Scientific, USA). Equivalent amounts of proteins were separated by SDS-PAGE electrophoresis, followed by transfer to PVDF membranes (Millipore, Darmstadt, Germany). Then, proteins were incubated with primary antibodies at 4 °C overnight, and consecutively with the corresponding secondary antibodies (1:5000; Boster, Wuhan, China) for 2 h at 37 °C. Finally, immunoblots were evaluated using a SynGene G: Box Chemi XRQ system (Alpha Metrix Biotech, Rödermark, Hesse, Germany).

### Immunofluorescence assay

The NPC cells were plated in 24-well plates pre-covered with sterile coverslips. After 8 Gy RT treatment, cells were fixed and permeabilized with 4% paraformaldehyde and 0.1% Triton X-100, respectively. Then, following blockage with 5% BSA coverslips were incubated with primary antibodies according to the protocol. Secondary antibodies were added at 37 °C for 2 h, followed by staining with DAPI. Finally, slides were observed under a laser scanning confocal microscope (Zeiss, Germany) and images were captured. Captured images were analyzed by Image J 1.8.0.

### Co-immunoprecipitation

Co-immunoprecipitation (Co-IP) experiments were performed using the Co-IP kit (absin, Shanghai, China) according to the manufacturer's protocol. Anti-flag antibodies and anti-p53 antibodies were used to form an immune complex with the target in the cell lysate. After incubation with protein A, and G for 12 h, the immunoprecipitates were analyzed by western blotting.

### Apoptosis analysis

Following irradiation with 8 Gy, cells were resuspended and collected in sterile 10 ml EP tube. After being centrifuged with 1500 g for 5 min and washed with cold-PBS, Cells were stained with Annexin V-APC and PI, following the manufacturer's protocol. Samples were analyzed by flow cytometry using a LSRFortessa cell analyzer (BD Biosciences, San Jose, CA, USA). Obtained results were analyzed using FlowJo version 10.

### Tumor sphere-forming assay

After being plated in 24-well ultralow attachment plates (Corning, NY) at various densities, cells were cultured with Dulbecco modified Eagle's medium/F12 supplemented with 20 % BIT9500 serum substitute (STEMCELL Technologies, South San Francisco, CA). 20 ng/mL EGF (PeproTech, Rocky Hill, NJ), and 20 ng/mL recombinant basic fibroblast growth factor (PeproTech, Rocky Hill, NJ) were also added. The diameter of the formed sphere should reach 100 μm or more. Formed spheres were observed and counted under a microscope after 1-2 wk. Every experimental group was repeated 3 times.

### Animals

For *in vivo* studies, we followed the National Institutes of Health guide for the care and use of Laboratory animals (NIH Publications No. 8023, revised 1978). Five hundred thousand CNE2 and CNE2-shEGLN1 cells were injected into the flanks of female BALB/c nude mice. Mice were randomly divided into 4 groups: control, control-RT, shEGLN1, and shEGLN1-RT. The volume of growing tumors was measured every 3 days using vernier calipers. The following equation was used to calculate the volume of the tumor: Tumor volume = length × width × width / 2. When tumors reached a volume of 100-150 mm^3^, the tumor areas of CNE2-RT and CNE-shEGLN1-RT groups were irradiated with 8 Gy. When the volume of the control group reached 2000 mm^3^, animals were sacrificed. Half of the primary tumors were directly frozen in -80 °C, and half were fixed with formalin and embedded in paraffin for further analyses. Mice were kept in a specific pathogen-free (SPF) animal facility.

### Statistical analysis

GraphPad Prism 5.0 was used for Statistical analysis. All *in vitro* experiments were independently performed 3 times. Data were shown as mean ± SD. ANOVA tests or *t*-tests were used for analysis of statistical significance. A value of P <0.05 was considered statistically significant.

## Results

### Expression of EGLN1 in patients with NPC

**Figure 1:**
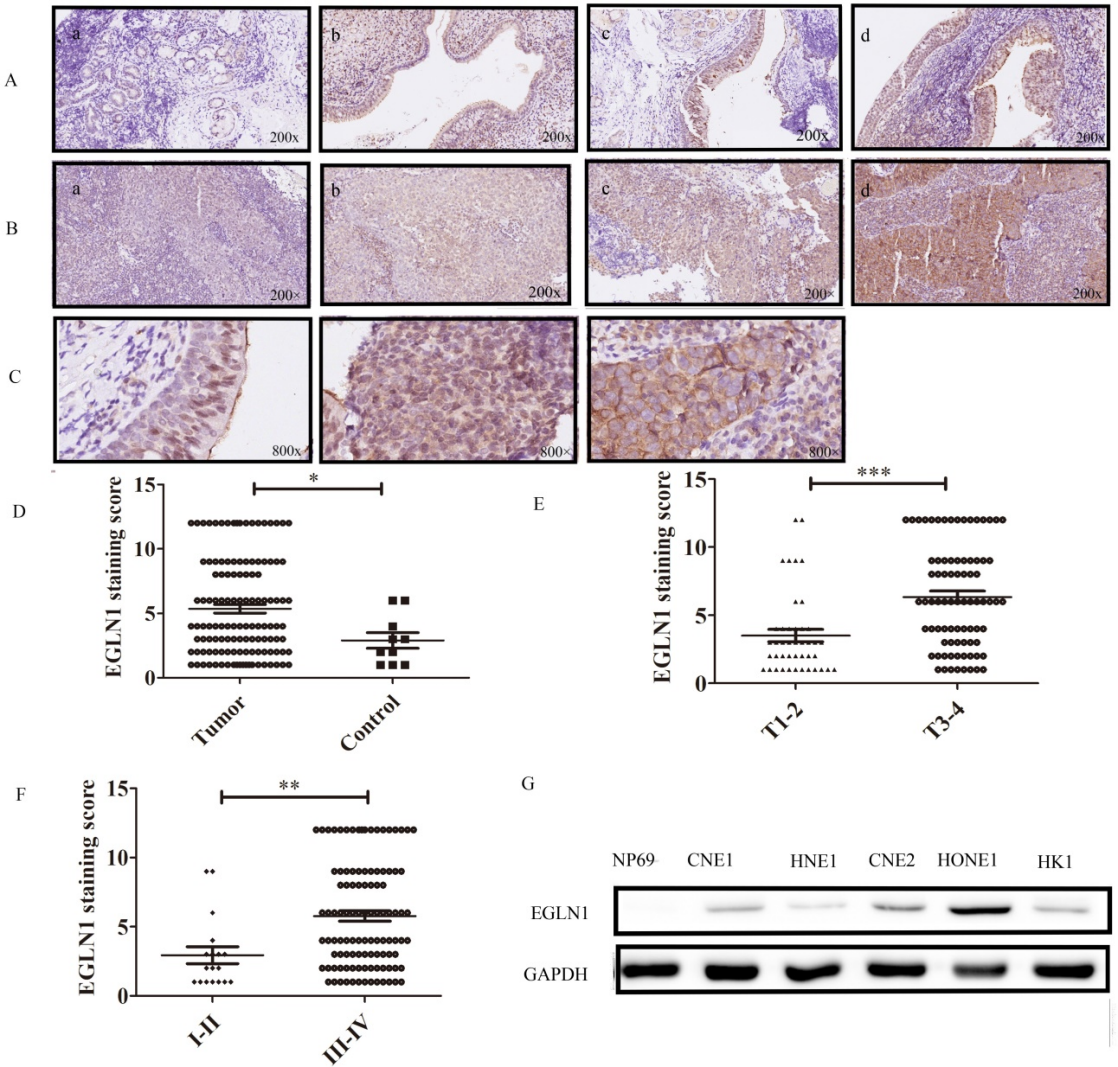
** Expression of EGLN1 in normal and NPC tissues and cells. A:** Representative IHC staining of EGLN1 in normal nasopharyngeal epithelium (×200); **B:** Representative IHC staining of EGLN1 in tissues of patients with NPC (×200); **C:** Differential subcellular localization of EGLN1 in different tissues (×800); **D-F:** Analysis of the varying expression of EGLN1 in relation to various clinical characteristics. **G:** Relative expression of EGLN1 in NP69 and NPC cell lines, determined by western blotting. Data are shown as means ± SEM. n = 124. *P < 0.05; **P < 0.01; ***P < 0.005.

Several studies have indicated that EGLN1 is expressed in endothelial cells. Accordingly, we confirmed that nasopharynx epithelium cells have different degrees of EGLN1 expression (Fig. 1A, n=10). Interestingly, EGLN1 could be detected in both the nucleolus and cytoplasm. However, the subcellular localization of EGLN1 showed heterogeneity in different individuals (Fig. 1C). We next detected the expression of EGLN1 in patients with NPC and presented the representative images in Figure 1B (n = 124). Normal tissues were characterized by a total of 10% (1/10) positive expression of EGLN1, whereas there was a total of 54.8% (68/124) high expression observed in patients with NPC. Moreover, compared with normal epithelial cells, cancer tissues exhibited a relatively high level of expression of EGLN1 (2.100 ± 0.3145 vs 5.363 ± 0.3365, P = 0.007; Table 1, Fig. 1D). In addition, T3-4 classification displayed a stronger staining score than T1-2 classification (6.346 ± 0.4169 vs 3.512 ± 0.4555, P < 0.0001; Table 1, Fig. 1E). Patients in stages I-II showed a lower expression of EGLN1 than those in stages III-IV (2.944 ± 0.6075 vs 55.774 ± 0.3660, P = 0.0027; Table 1, Fig. 1F). Based on these findings, it was suggested that high T classification and high clinical stages were characterized by a high level of expression of EGLN1. However, the expression of EGLN1 was not observed to be correlated with patient gender, age, distant metastasis and N classification (Table 1). We further compared the expression levels of EGLN1 in NPC cell lines with normal endothelial cells (NP69). The NPC cell lines showed the higher expression of EGLN1 than that in normal human nasopharyngeal epithelium cells (Fig. 1G).

### EGLN1 promoted cell proliferation, migration and invasion *in vitro*

**Figure 2:**
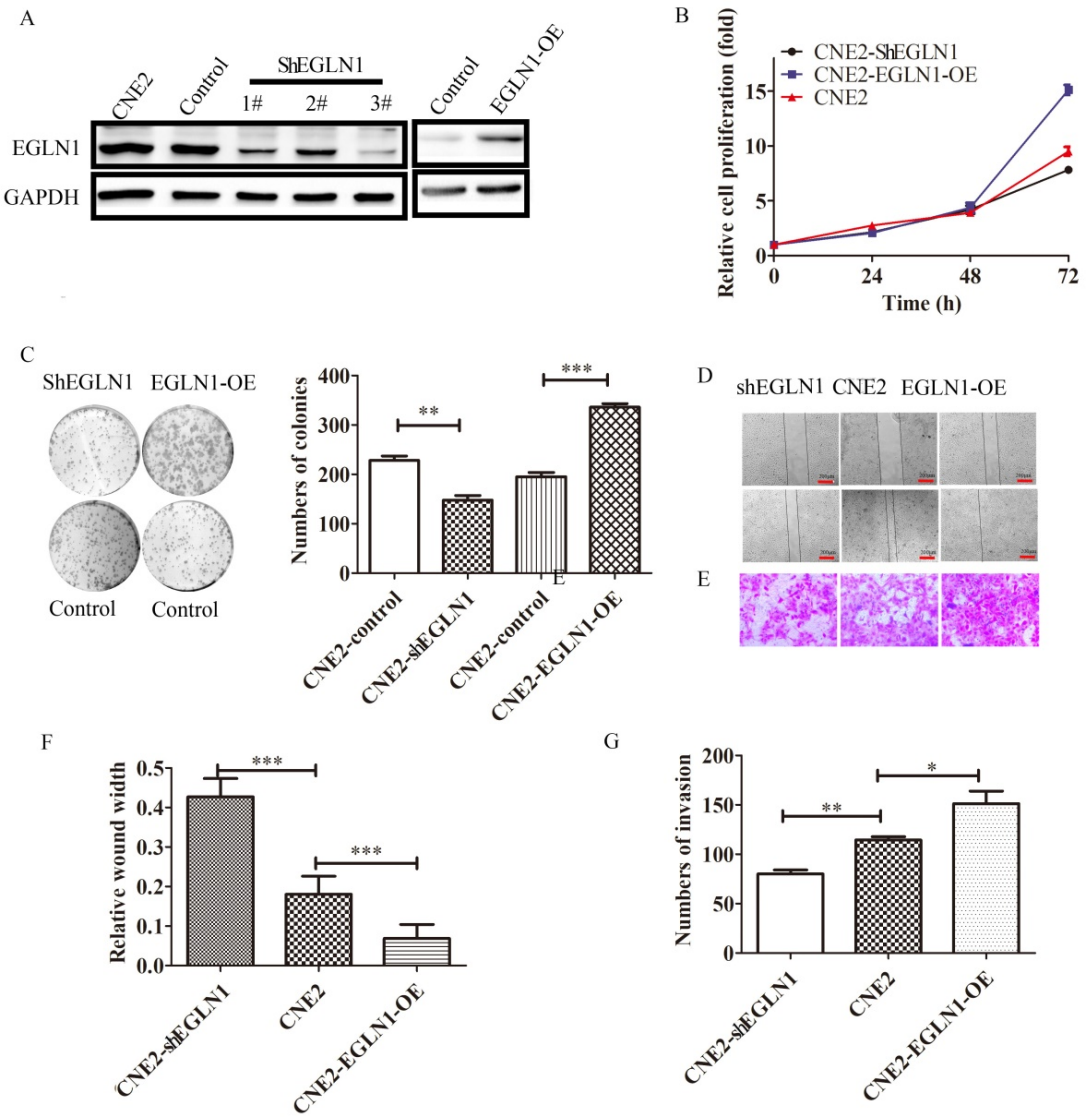
** The effect of the expression of EGLN1 on cell proliferation, migration, and invasion. A:** CNE2 cells were transfected with shEGLN1 or EGLN1-OE lentiviral constructs, and the expression of EGLN1 was analysed by western blotting. **B:** The proliferation ability was measured by CCK8 assays. Overexpression of EGLN1 promotes the proliferation of CNE2, whereas silencing of EGLN1 suppresses it. **C:** EGLN1 can regulate the ability of cell clone formation. **D:** The migration ability of NPC cells was examined by scratch assays. Images of the scratch were captured at 0 and 24 h. **E:** The invasion of NPC cells was measured by transwell invasion assay. Images were captured at 24 h. F, **G:** Data were analyzed. Data are presented as means ± SEM corresponding to three independent experiments. **P < 0.01; ***P < 0.005.

In order to investigate the biological function of EGLN1 in the progression of NPC, EGLN1-OE (overexpression) and shEGLN1 lentiviral vectors were stably transfected into CNE2 cells. The efficiency of the overexpression and knockdown constructs was detected by western blot analysis (Fig. 2A). EGLN1 was shown to significantly promote the proliferation of NPC cells (Fig. 2B). Overexpression of EGLN1 showed a positive effect on colony formation (195.3 ± 8.413 vs 336.0 ± 7.095, p<0.01), whereas silencing of EGLN1 led to the opposite effect (228.3±8.838 vs 147.7 ± 9.528, p<0.001, Fig. 2C). In addition, the ability of NPC cells for migration and invasion was also tested using the scratch tests and transwell assays (Fig. 2D, E). In scratch tests, the relative wound width of CNE2-EGLN1-OE cells was significantly decreased compared to the control group (0.4267 ± 0.04667 vs 0.1800 ± 0.04619, p<0.001). Contrarily, the relative wound width of CNE2-shEGLN1 (0.06863 ± 0.03535, p<0.001) was significantly increased (Fig. 2F). In Transwell assays, the number of invasion cells was increased in CNE2-EGLN1-OE group (151.3 ± 12.57, p<0.01) and decreased in CNE2-shEGLN1 group (80.00 ± 4.163, p<0.05) compared with control (114.3 ± 3.480). These results indicated that overexpression of EGLN enhanced the migration and invasion ability of CNE2, whereas the loss of EGLN1 reduced it (Fig. 2G).

### EGLN1 enhanced the stemness potential of NPC

**Figure 3:**
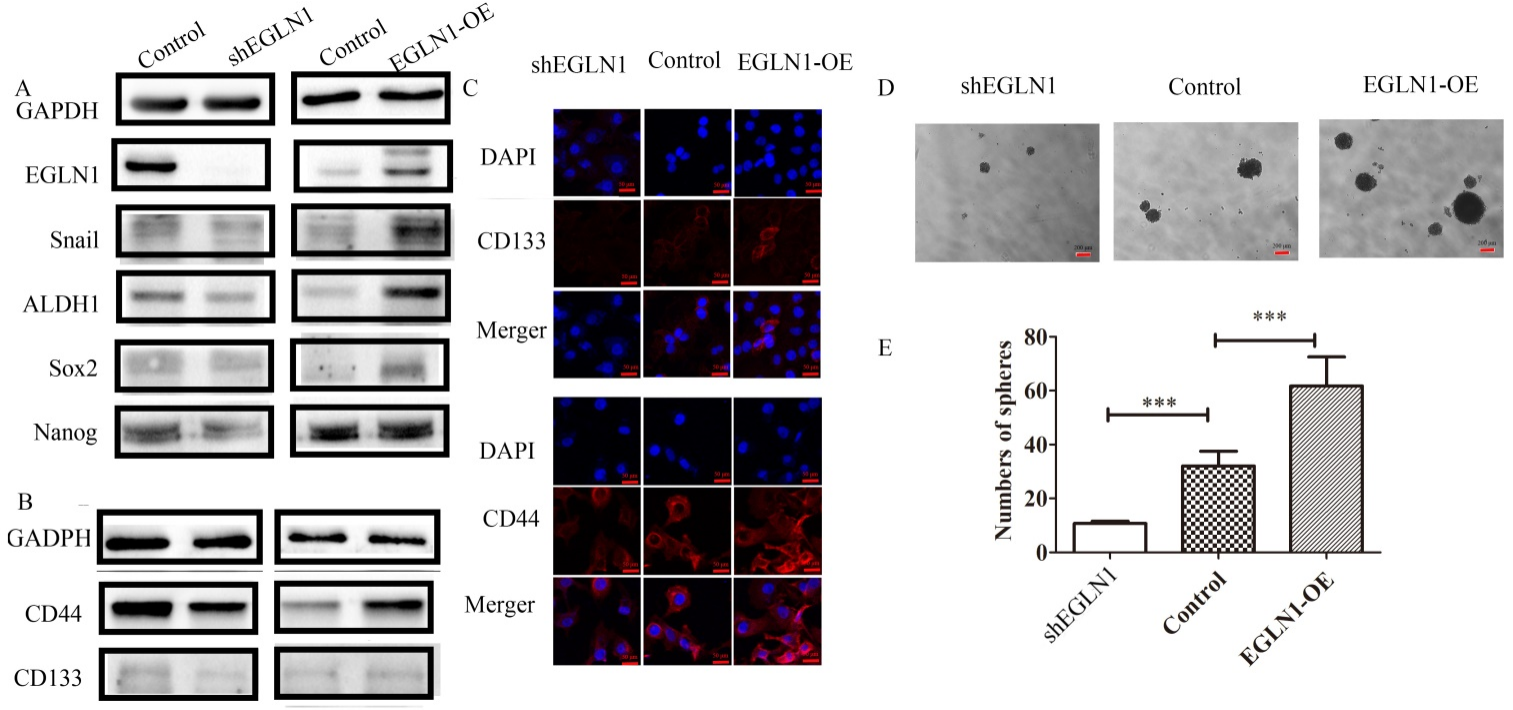
** EGLN1 increases the CSC-like phenotype in CNE2. A:** The expression of the CSC-associated transcription factors Nanog, Sox2, Snail, and ALDH1 was detected by western blot analysis. **B:** The expression of CD44 and CD133 was tested by both western blotting and immunofluorescence. **C:** EGLN1 enhanced the expression of CD44 and CD133. **D, E:** EGLN1 enhanced the formation of spheres in CNE2. Data are presented as means ± SEM corresponding to three independent experiments. ***P < 0.005.

EGLN1 has been suggested to play a pivotal role in the initiation and progression of NPC. To investigate the role of EGLN1 in cancer cell self-renewal and differentiation, we detected the effect of EGLN1 on the CSC properties of NPC cells. As shown in Figure 3A, EGLN1 mediated gene transcription, regulating the subsequent plasticity of CSCs. In our study, Nanog homeobox (NANOG), SRY-box transcription factor 2 (SOX2), and aldehyde dehydrogenase 1 (ALDH1) were upregulated in EGLN1-OE groups and downregulated in the shEGLN1 group. Furthermore, we examined the expression of the CSC surface markers, CD133 and CD44, by western blotting and immunofluorescence (Fig. 3B, C). The expression of both CD133 and CD44 was enhanced in EGLN1-OE cells, whereas it was reduced in ShEGLN1 cells. Thus, EGLN1 might increase the rate of CD44+/CD133+ CSC-like cells in NPC cell lines. Accordingly, the CSC-like phenotype of NPC cells was examined by performing the sphere formation assay (Fig. 3D, E). The number of spheres cells was significantly increased in CNE2-EGLN1-OE group (61.67±10.81, p<0.001) and decreased in CNE2-shEGLN1 group (10.67 ± 0.8819, p<0.01) compared with control (32.00 ± 5.5080). As we expected, EGLN1 promoted the capability of tumor sphere formation of CNE2. Similarly, silencing of EGLN1 was shown to reduce the ability for tumor sphere formation.

### EGLN1 promoted radioresistance in NPC both *in vitro* and *in vivo*

**Figure 4:**
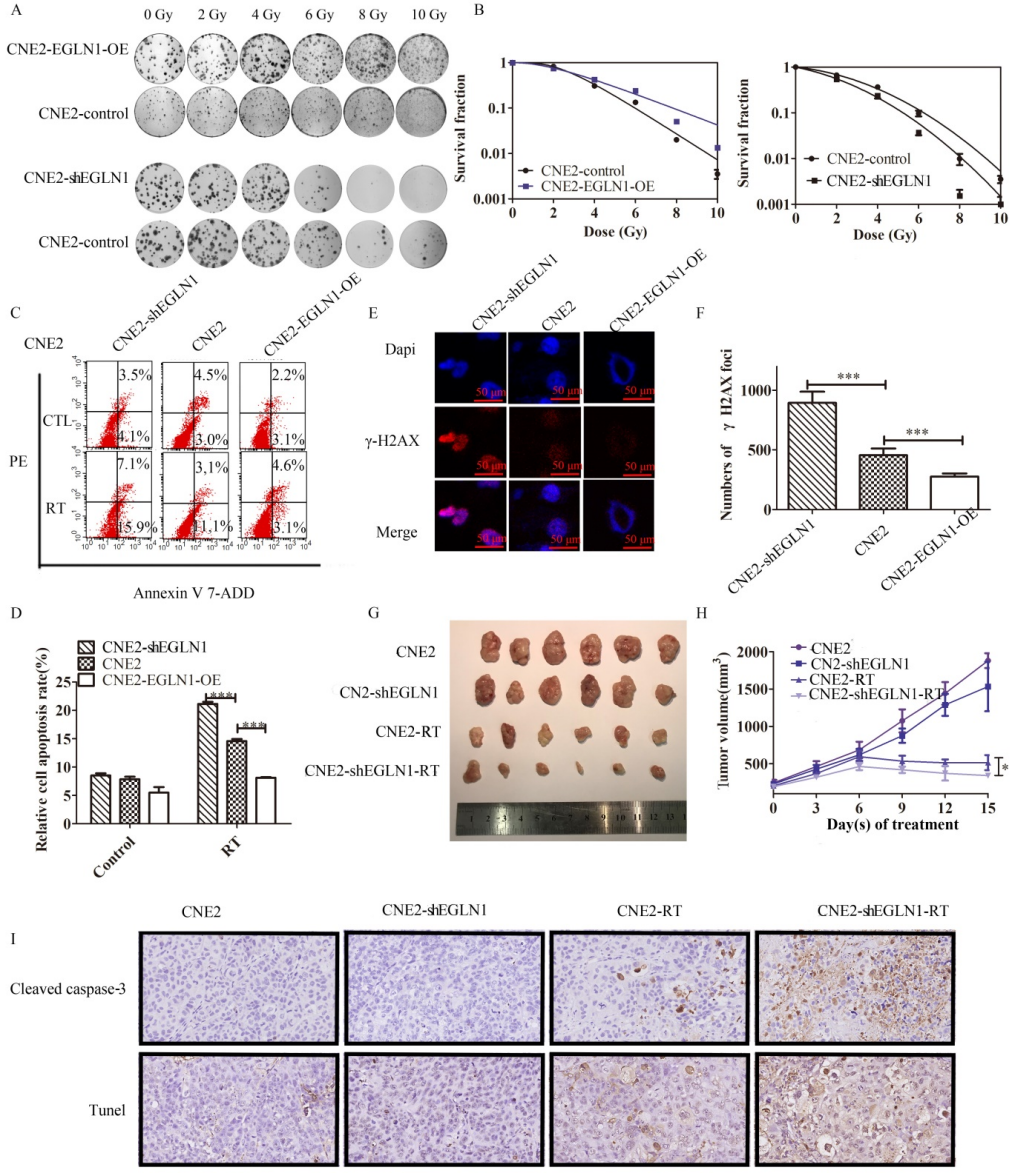
** EGLN1 inhibits the response to irradiation in CNE2 both *in vitro* and *in vivo*. A,B:** To analyze the effect of EGLN1 on the survival of NPC cells after exposure to 6 single-radiation doses (0, 2, 4, 6, 8, and 10 Gy), their clonogenic ability was assessed. Quadratic and linear model equations were proposed for the analysis of radioresistance. **C,D:** Cell apoptosis in different groups was detected by flow cytometry analyses. Apoptosis rates decreased in EGLN1-OE groups and increased in ShEGLN1 groups compared with control. **E, F:** γH2AX was stained red, and the cell nucleus with DAPI (blue). The number of γH2AX was counted by analyzing 100 randomly selected cells. **G:** Representative tumor xenografts. **H:** Tumor volumes were measured every 3 d. **I:** Representative immunohistochemical images of caspase 3 detection and tunel assay in different groups are shown (×200). Data are presented as means ± SEM corresponding to three independent experiments. *P < 0.05; ***P < 0.005.

In our previous study, we demonstrated that the radioresistance of NPC also depended on the presence of CSCs, compared with normal cancer cells, CSCs may facilitate DNA repair 21. CNE2 cells transfected with either EGLN1-OE or ShEGLN1 lentiviral vectors were exposed to increasing doses of radiotherapy (RT; 0-10 Gy). Silencing of EGLN1 resulted in decreased number of CNE2 colonies compared to the control group. On the other hand, overexpression of EGLN1 in CNE2 cells increased the radioresistance compared with the control group as shown in the colony assay (Table 2, Fig. 4A, B). Cell apoptosis was decreased in CNE-EGLN1-OE group (8.111 ± 0.1119, p<0001) and increased in CNE2-shEGLN1 group (21.14 ± 0.3826, p<0001) compared with control group (14.56 ± 0.3837) after RT. The level of expression of EGLN1 in NPC cells might have a negative correlation with cell apoptosis after radiotherapy (Fig. 4C, D). It is well known that RT induces cell apoptosis mainly through the generation of DNA double strand breaks (DSBs). By performing immunoprecipitation, we detected the presence of γ-H2AX, the marker of DSBs. Compared with the control group (454.0 ± 27.77), the number of γ-H2AX was higher in the shEGLN1 group (894.7 ± 94.31, p<0.01), but lower in the EGLN1-OE group (277.7 ± 25.71, p<0.01) following 8 Gy irradiation to cells (Fig. 4E, F). We further explored the role of EGLN1 *in vivo*. We injected CNE2 and CNE2-shEGLN1 cells into BALB/c nude mice and measured the growth of the generated tumors every 3 d. Mice were randomly divided into 4 groups: control, RT, ShEGLN1, and shEGLN1-RT. Although, shEGLN1 xenografts (1215 ± 65.3) grew smaller than the control ones (1442 ±152.3), there was no statistical difference observed between them. The RT and ShEGLN1-RT groups were treated with 8 Gy irradiation when tumor size reached 100-150 mm^3^. Consistent with our *in vitro* results, the growth of tumors in both treated groups was hindered (613.4±77.72 vs 309.7±34.74, p<0.05, Fig. 4G, H). In addition, immunohistochemistry and Tunel assay were performed to detect cleaved caspase 3 (CASP3) and evaluate apoptosis in cells, respectively. As presented in Figure 4I, the rate of apoptosis was higher in the CNE2-shEGLN1 group compared with CNE2 alone, especially after RT.

### p53 was regulated by EGLN1 via the hydroxylation pathway

**Figure 5:**
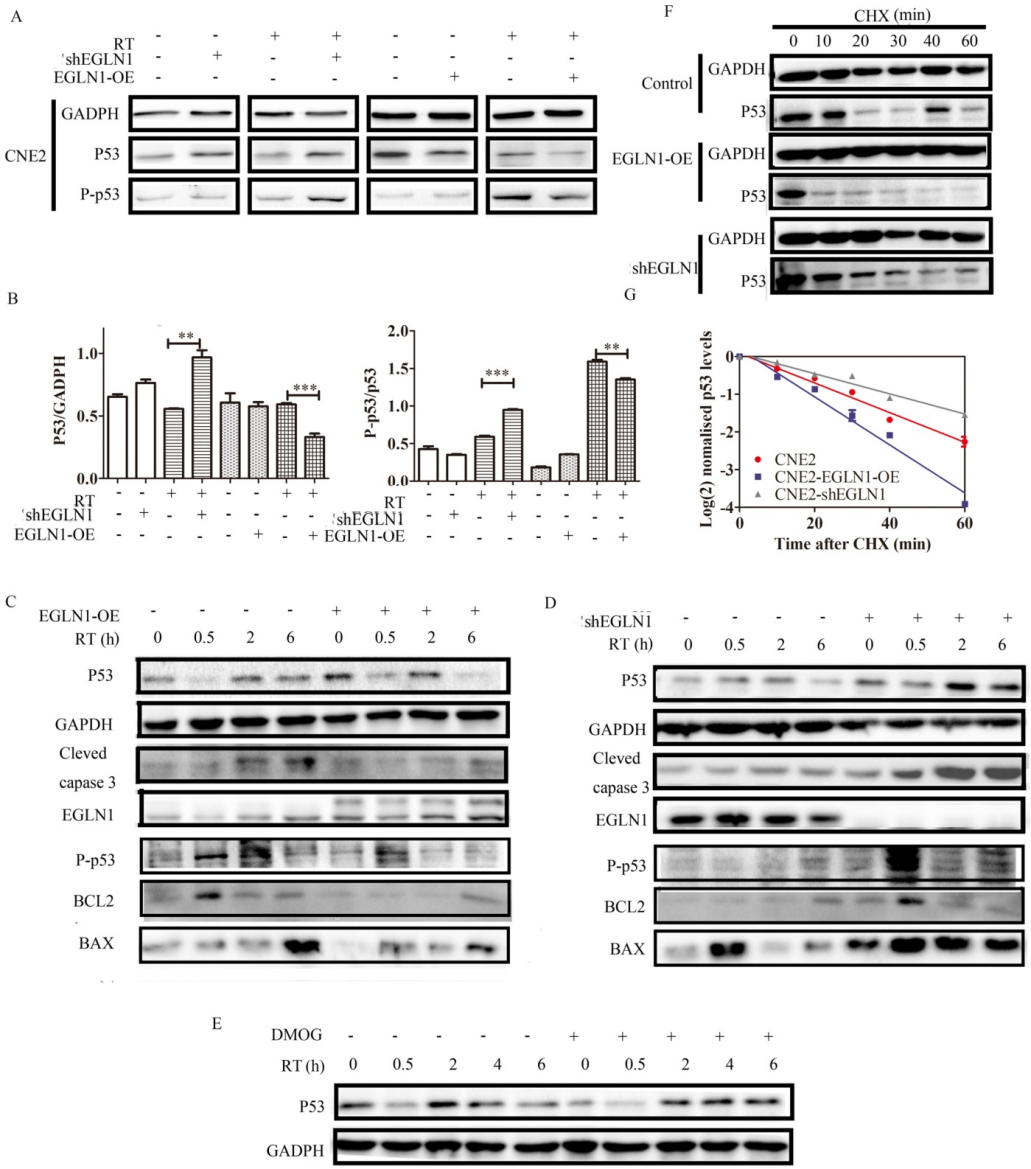
** EGLN1 regulates p53 via its hydroxylation activity. A,B:** CNE2 cells were transfected with either an EGLN1-overexpressing, an EGLN1-shRNA, or a corresponding non-targeting vector (control). Subsequently, p53 was detected in cells treated with RT or not. **C,D:** The downstream cascades of p53 were detected by western blotting. **E:** CNE2 irradiated with 8 Gy prior to incubation with 2.5 mM DMOG or not. Protein lysates were analyzed. **F,G:** Cells were incubated with 2.5 mM DMOG or DMSO for 4 h and followed by treatment with or without CHX (10 µg/mL) for up to 8 h. The relative intensity of p53 was plotted on a XY diagram to visualize the half-life of the protein. Data are presented as means ± SEM corresponding to three independent experiments.

As shown, EGLN1 was associated with clinical stages, tumor formation, self-renewal, and differentiation. Moreover, EGLN1 could induce tumor resistance to radiation. However, the underlying mechanism governing these effects remained unclear. We found that overexpression of EGLN1 in the CNE2 NPC cell lines could reduce the protein levels of p53, especially after radiotherapy (RT). In contrast, silencing of EGLN1 was shown to enhance the expression of p53, consistent with the results above (Fig. 5A, B). In addition, we found that downstream cascades, such as the levels of BAX, BCL2 and other apoptosis-related proteins were decrased in EGLN1-OE cells after RT. After knockout, apoptosis-related proteins were increased (Fig. 5C, D). Incubation of CNE2 cells with DMOG, a pan hydroxylase inhibitor, resulted in higher levels of p53 protein relative to control (Fig. 5E). To verify whether the increasing levels of p53 were the result of post-transcriptional regulation, we incubated cells with cycloheximide (CHX), which can block the elongation phase of eukaryotic translation. We noted that the protein level of EGLN1 shortened the half-life of p53 (Fig. 5F-G).

### EGLN1 regulated the stability and ubiquitination of p53 protein

**Figure 6:**
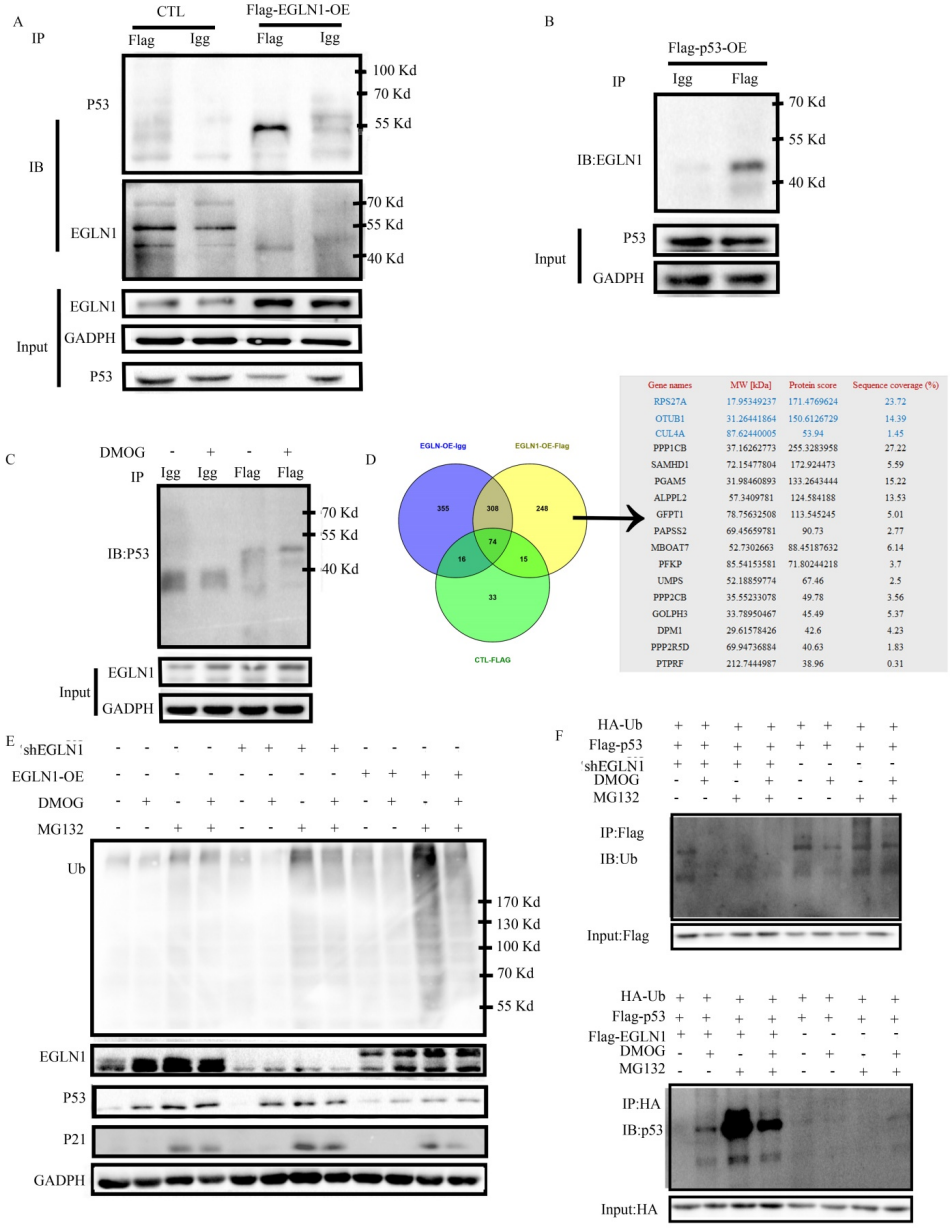
** EGLN1 regulates p53 via the ubiquitination system. A:** CNE2 cells were transfected with Flag-EGLN1, Flag-EGLN1 was immunoprecipitated and analyzed by western blotting. EGLN1 interacted with p53. **B:** Flag-p53 was transfected into CNE2. p53 can bind with EGLN1. **C:** DMOG could enhance this interaction. **D:** Differential proteins were examined by LC-MS/MS analysis and analyzed by Venny2.0.2. **E:** Total level of ubiquitination was analyzed. **F:** IB analysis of pull-down products derived from cells transfected with constructs encoding indicated proteins and treated with treated with MG132, or DMOG, or both for 4 h using an anti-ubiquitin antibody.

To verify whether EGLN1 interacts with p53, we transfected CNE2 cells with Flag-EGLN-OE, or Flag-P53-OE. A control group was transfected with an empty vector. The Flag immunoprecipitated proteins were detected by western blotting. As presented in Figure 6A, and 6B, we identified that EGLN1 could bind with endogenous p53 protein in normoxia, and vice versa. Interestingly, DMOG increased the interaction between them (Fig. 6C). In order to further clarify the role of hydroxylation of p53 by EGLN1, we identified the Flag immunoprecipitate proteins by LC-MS/MS analysis. The differential protein profile was analyzed by Venny 2.0.2 (Fig. 6D). The EGLN1-P53 complex could bind with ubiquitinating and phosphorylation enzymes (Table 3). At first, we observed that there was an increase in the levels of p53 when cells were exposed to DMOG, and this effect could be further increased following treatment with the MG132 ubiquitinate inhibitor. We then tested the effect of EGLN1 on the total ubiquitination level in CNE2 cells. The total ubiquitination level was increased in EGLN1-overexpressing cell lines, whereas EGLN1 knockout and incubation with DMOG reduced it (Fig. 6E). In Figure 6F, we confirmed that endogenous ubiquitination of p53 could be reduced by DMOG. We pulled down Flag-p53 or HA-Ub in differenent cells incubated with or without MG132 and DMOG and discovered that the ubiquitination of p53 was decreased in shEGLN1 cells or under exposure to DMOG and increased in EGLN1-OE cells. These results suggested that EGLN1 could bind to P53 and regulate the stability and ubiquitination of p53 protein.

## Discussion

The function of EGLN1 and its intrinsic mechanism in tumors remains controversial and unclear. We demonstrated in this study that the expression level of EGLN1 was upregulated in T3-4 compared with T1-2 classification tissues. In addition, we also found that the cellular localization of EGLN1 was not consistent in different individuals. In some patients, EGLN1 was mainly distributed in the cell nucleus, whereas in others it was found in the cell cytoplasm. Studies have been reported that T3-4 stage tumors have a poorer prognosis compared with T1-2 tumors. Thus, high expression of EGLN1 might function as a poor prognostic factor of patients with NPC. However, there are also some limitations regarding to our study, owing to the small sample size. Furthermore, extended survival analysis are needed to be accomplished in the future. In addition, we did not investigate the differences in the subcellular localization of EGLN1 and the reasons behind them. Back to 2006, Terhi Jokilehto and Krista Rantanen reported that the expression of EGLN1 increased in the less differentiated phenotype, partially relocating from cytoplasm to nucleus in HNSCC. They thus proposed that EGLN1 regulated tumor aggressiveness independent of the HIF1α pathway 22. We also found that EGLN1 could enhance oncogenesis and increase the ability of cell renewal, invasion, and migration in our *in vitro* experiments.

In addition to discovering the value of EGLN1 in predicting prognosis and its role in the physiological function of NPC cells, we further explored the underlying mechanism that regulated its function in NPC. We showed in this study that EGLN1 promoted ubiquitination and degradation of p53, and inhibited its phosphorylation, through direct interaction with p53, via its hydroxylase activity. These results seemed to be contrary to the regulation of p53 by other members of its family. Previous studies have reported that EGLN2 and EGLN3 could stabilize p53 and activate the associated downstream pathways. EGLN3 could bind to MDM2, which acts as an E3 ubiquitin ligase in the p53 degradation system. Thus, EGLN3 was reported to lead to the upregulation of the activity of p53, and maintainance of its activation 23,24. In addition, EGLN2 was shown to promote the activation of p53 after chemotherapy in colorectal cancer cell lines, thereby promoting DNA damage repair and inhibiting cell death. Therefore, EGLN2 might be part of the reason for the observed chemotherapy resistance to CRC 24. However, in our study, our data suggested a negative correlation between EGLN1 and p53 and the downstream pathway. EGLN1 and p53 could interact with each other. DMOG might decrease the degradation of the enzyme-substrate complex. We found that the regulation of p53 by the DMOG hydroxylase inhibitor was reversed when EGLN1 was overexpressed and accompanied by an increase in the levels of ubiquitination. These results indicated that the hydroxylase activity of EGLN1 might lead to an increase in proteasomal degradation via ubiquitination. We believe that p53 can potentially be a hydroxylase substrate for EGLN1. So, EGLN1 could downregulate the expression of p53 through the ubiquitination system in a manner different from that exhibited by other family members.

It is worth noting that p53 is known to be a tumor suppressor which inhibits oncogenesis and self-renewal during tumorigenesis, posing an obstacle to the formation of CSCs 25. Cancer stem cells are defined as a population of cancer cells with the ability of self-renewal and high capacity for tumorigenicity. CSCs have been identified in many solid tumors, including breast, pancreatic, ovarian, brain, colon, prostate, and liver cancers. Moreover, ALDH1, CD44, CD133, and NANOG were suggested as unique CSC markers 26. In our study, EGLN1 could suppress the expression of p53 and increase the expression of CSC factors, including CD44, CD133, NANOG, SOX2, and ALDH1. Thus, EGLN1 might act as a promotor for the formation of CSCs in NPC. In fact, our data showed that overexpression of EGLN1 led to increased CSC properties of NPC cells, whereas the contrary results were observed in EGLN1-silenced cells. It is well known that CSCs are resistant to conventional anti-tumor treatments, ultimately leading to therapy failure 21,27. A correlation between CSCs and radiotherapy resistance in NPC has already been reported in our previous study 21. In this study, our data suggested that overexpression of EGLN1 resulted in lower levels of DNA damage and decreased rate of apoptosis after radiation. At the same time, inhibition of the expression of EGLN1 led to better response to treatment and increased apoptosis after radiation. We also demonstrated that EGLN1 could decrease DSBs and cell apoptosis, thus resulting in increased radioresistance in NPC cells. In addition, our study indicated that EGLN1 could enhance radioresistance of NPC cells *in vivo*. This finding indicated that EGLN1 might play a role in the response to RT through p53. Hence, EGLN1 might act as a promising therapeutic target for patients with NPC.

Taken all together, our findings demonstrated the close correlation among EGLN1, p53, the properties of CSCs, and radioresistance. We found that EGLN1 could function as a poor prognostic factor in patients with NPC. EGLN1 could downregulate p53 and inhibit its activation though the ubiquitin-proteasome system, depending on its hydroxylase activity. EGLN1 appears to be able to enhance the properties of CSCs in NPC cell lines by degrading p53, which could be the major reason for radioresistance.

## Figures and Tables

**Table 1:** The semiquantitative immunohistochemical score of EGLN1 expression in noncancerous and cancerous tissues and the clinic‐pathological characteristics of NPC patients

Characteristics	Mean ± SEM	No. of patients	P‐value
Noncancerous tissues	2.100±0.3145	10	0.007**
Cancerous tissues	5.363± 0.3365	124	
**Age**			
<60	5.296±0.3761	98	0.5659
≥60	5.769±0.7344	26	
**Gender**			
Male	5.221 ± 0.3947	86	0.5278
Female	5.684± 0.6435	38	
**T classification**			
T1‐2	3.512± 0.4555	43	< 0.0001***
T1	4.167± 0.8679	18	
T2	3.040 ± 0.4672	25	
T3‐4	6.346 ± 0.4169	81	
T3	5.800± 0.6100	40	
T4	6.878± 0.5644	41	
**N classification**			
N0‐1	5.087 ± 0.5355	46	0.531
N0	5.438± 0.9485	16	
N1	4.900± 0.6563	30	
N2‐3	5.526± 0.4333	78	
N2	5.782± 0.5367	55	
N3	4.913± 0.7166	23	
**M classification**			
M0	5.286± 0.3546	112	0.4856
M1	6.083± 1.090	12	
**Clinical stage**			
I‐II	2.944± 0.6075	18	0.0027**
I	4.333± 2.404	3	
II	2.667± 0.5828	15	
III‐IV	5.774± 0.3660	106	
III	5.109± 0.5623	46	
IV	6.283± 0.4754	60	

**Table 2:** The radiobiological parameters of NPC cells exposed to radiation

	SF_2_	D_0_ (Gy)	Dq (Gy)	N	SER
CNE2-Control	0.67	2.04	1.87	2.49	-
CNE2-shEGLN1	0.54	1.76	1.30	2.09	1.16
CNE2-Control	0.71	1.51	2.57	5.47	-
CNE2-EGLN1-OE	0.82	2.48	2.19	2.42	0.61

**Table 3:** Differential genes were analyzed by Venny 2.0.2

Gene names	MW[kDa]	Protein score	Sequence coverage (%)
RPS27A	17.953492	171.4769624	23.72
0TUB1	31.26441864	150.6126729	14.39
CUL4A	87.62440005	53.94	1.45
PPP1CB	37.16262773	255.3283958	27.22
SAMHD1	72.15477804	172.924473	5.59
PGAM5	31.98460893	133.2643444	15.22
ALPPL2	57.3409781	124.584188	13.53
GFPT1	78.75632508	113.545245	5.01
PAPSS2	69.45659781	90.73	2.77
MBOAT7	52.7302663	88.45187632	6.14
PFKP	85.54153581	71.80244218	3.7
UMPS	52.18859774	67.46	2.5
PPP2CB	35.55233078	49.78	3.56
GOLPH3	33.78950467	45.49	5.37
DPM1	29.61578426	42.6	4.23
PPP2R5D	69.94736884	40.63	1.83
PTPRF	212.7444987	38.96	0.31

## Abbreviations

ALDH1: aldehyde dehydrogenase 1
CSCs: cancer stem cell
MG132: carbobenzoxy-Leu-Leu-leucinal
CRT: chemoradiotherapy
Co-IP: co-immunoprecipitation
CHX: cycloheximide
DMOG: dimethyloxalylglycine
DSBs: double strand breaks
EGLN1: Egl-9 Family Hypoxia Inducible Factor 1
FBS: fetal bovine serum
HIF1α: hypoxia-inducible factor 1α
IMRT: intensity-modulated radiotherapy
NANOG: Nanog homeobox
NPC: nasopharyngeal carcinoma
PVDF: polyvinylidene fluoride
PFS: progression-free survival
PHD2: prolyl Hydroxylase Domain 2
PKB: protein kinase B
RT: radiotherapy
SPF: specific pathogen-free
HNSCC: squamous cell cancer of the head and neck
SOX2: SRY-box transcription factor 2
VHL: von Hippel-Lindau
